# Preoperative imaging findings in patients undergoing transcranial magnetic resonance imaging-guided focused ultrasound thalamotomy

**DOI:** 10.1038/s41598-021-82271-8

**Published:** 2021-01-28

**Authors:** Cesare Gagliardo, Roberto Cannella, Giuseppe Filorizzo, Patrizia Toia, Giuseppe Salvaggio, Giorgio Collura, Antonia Pignolo, Rosario Maugeri, Alessandro Napoli, Marco D’amelio, Tommaso Vincenzo Bartolotta, Maurizio Marrale, Gerardo Domenico Iacopino, Carlo Catalano, Massimo Midiri

**Affiliations:** 1 Department of Biomedicine, Neuroscience and Advanced Diagnostics, University of Palermo, Via del Vespro 129, 90127 Palermo, Italy; 2 Department of Physics and Chemistry, University of Palermo, Palermo, Italy; 3 Department of Radiological, Oncological and Anatomopathological Sciences, ‘Sapienza’ University of Rome, Rome, Italy

**Keywords:** Diseases of the nervous system, Neuroscience, Medical research, Movement disorders, Multiple sclerosis, Neuropathic pain, Parkinson's disease, Neurological disorders

## Abstract

The prevalence and impact of imaging findings detected during screening procedures in patients undergoing transcranial MR-guided Focused Ultrasound (tcMRgFUS) thalamotomy for functional neurological disorders has not been assessed yet. This study included 90 patients who fully completed clinical and neuroradiological screenings for tcMRgFUS in a single-center. The presence and location of preoperative imaging findings that could impact the treatment were recorded and classified in three different groups according to their relevance for the eligibility and treatment planning. Furthermore, tcMRgFUS treatments were reviewed to evaluate the number of transducer elements turned off after marking as no pass regions the depicted imaging finding. A total of 146 preoperative imaging findings in 79 (87.8%) patients were detected in the screening population, with a significant correlation with patients’ age (rho = 483, p < 0.001). With regard of the group classification, 119 (81.5%), 26 (17.8%) were classified as group 1 or 2, respectively. One patient had group 3 finding and was considered ineligible. No complications related to the preoperative imaging findings occurred in treated patients. Preoperative neuroradiological findings are frequent in candidates to tcMRgFUS and their identification may require the placement of additional no-pass regions to prevent harmful non-targeted heating.

## Introduction

Transcranial Magnetic Resonance imaging-guided Focused Ultrasound (tcMRgFUS) is a novel incisionless stereotactic procedure based on the thermal ablation of a brain target using a high-intensity focused ultrasound (HI-FU) beam guided by MR anatomical imaging and thermal maps^1^. The clinical applications of tcMRgFUS are rapidly expanding and the procedure is nowadays indicated for the treatment of medication-refractory essential tremor, idiopathic unilateral tremor-dominant Parkinson’s disease, and neuropathic pain^2^. Randomized controlled trials and multiple studies have demonstrated the clinical benefits of tcMRgFUS for the treatment of movement disorders, with significant improvement in tremor scales and quality of life^3–9^. Moreover, experimental studies have explored the potential use of low intensity focused ultrasound (LI-FU) tcMRgFUS for transient blood brain-barrier opening in intracranial tumors^10–13^ and neurodegenerative disorders^14–17^. Considering movement disorders treatments, tcMRgFUS has demonstrated similar clinical efficacy and higher safety compared to other stereotactic procedures such as deep brain stimulation, radiofrequency ablation, or stereotactic radiosurgery due to the lack of ionizing radiations, craniotomy, or hardware-related complications^18–22^.

Rapid expansion in clinical applications poses a question regarding the clinical relevance of neurological conditions and neuroradiological imaging findings detected during screening procedures. Indeed, all candidates to tcMRgFUS procedures must undergo CT and MRI for treatment planning and simulation. During a tcMRgFUS procedure, the HI-FU waves emanating from the helmet-like transducer must travel through multiple tissue layers on a large head area prior to reaching the desired thalamic target. The acoustic properties and the thickness of each single tissue layer will influence the amount of energy carried by the acoustic wave that will be transmitted rather than reflected or absorbed. The energy absorption may result in unwanted thermal exposures. Thus, is mandatory to identify energy absorbing or sensitive structures on neuroradiological examinations to block the passage of the HI-FU beams through.

Nowadays only general contraindications to tcMRgFUS procedures are reported in the “Information for Prescribers” available from the vendor^23^. However, candidates to tcMRgFUS may present incidental findings on screening imaging studies that may influence the passage of the ultrasound beam or significantly impact the number of active transducer elements for the procedure. Prior studies have reported how common incidental brain findings on MRI are in the general population^24–27^. Once excluded any potentially clinically relevant incidental finding requiring urgent or immediate referral, which in fact would represent an absolute contraindication to the treatment, there are no precise indications about other incidental neuroradiological findings. To our knowledge, no prior study has assessed the implications of incidental neuroradiological findings and the role of preoperative imaging in tcMRgFUS treatment.

Therefore, the purpose of this study was to assess the prevalence and significance of imaging findings detected during screening procedures in candidates to tcMRgFUS thalamotomy and to determine their impact for the tcMRgFUS treatment eligibility.

## Materials and methods

The institutional review board (“Comitato Etico Palermo 1”) approved this study. All subjects provided written informed consent before enrolling for tcMRgFUS in accordance with the Declaration of Helsinki. This study is a retrospective analysis of prospectively-enrolled cohort undergoing clinical and neuroradiological screening for tcMRgFUS.

### Population

The study population included all consecutive adult patients prospectively enrolled for tcMRgFUS screening procedures between January 2015 and December 2019. A total of 298 patients underwent clinical screening as potential candidates for tcMRgFUS. In our University Hospital all the potential candidates were clinically evaluated by neurologists (specialized in movement disorders) and neurosurgeons (with expertise in conventional functional neurosurgery techniques) in order to confirm the clinical indication for the treatment. Clinical approved (CE marking of conformity no 2110597CE01) indications for treatment were medical-refractory essential tremor, idiopathic asymmetrical tremor-dominant Parkinson’s disease, and neuropathic pain. A few patients with different clinical conditions have been evaluated for experimental applications too (multiple sclerosis-associated tremor, dystonia, and vascular tremor). The Clinical Rating Scale for Tremor and the Essential Tremor Rating Assessment Scale were used for the clinical assessment of essential tremor patients and the Unified Parkinson’s Disease Rating for Parkinson disease patients^28–30^. At our center patients without clinical indication or with lack of definitive diagnosis were excluded and did not undergo screening imaging examinations. Patients considered suitable for tcMRgFUS underwent preoperative CT and MRI the same week of the clinical evaluations. CT was acquired in order to calculate the skull-density ratio (SDR). This score quantifies the discrepancy in Hounsfield Units between the spongious and cortical bone and it is mandatory to apply the phase correction algorithm for the HI-FU beams that allow a precise trans-cranial focusing and energy release on target^31,32^. CT is also used to identity frontal sinuses and calcifications that may interfere the HI-FU transmission. MRI was performed in order to plan the tcMRgFUS treatment simulation, to detect any preoperative imaging findings that may affect the procedure, and to delineate the optimal target coordinates^2^. Only patients that completed the clinical and neuroradiological screening were included for the present study.

### MRI technique

Patients that passed clinical screening underwent MRI screening using a 1.5 T MR scanner (Signa HDxt; GE Medical Systems). In our center the standard screening protocol included the following sequences: 2D fast recovery fast spin echo (FRFSE) T2-weighed (T2w) images (basal ganglia region coverage) acquired on sagittal, coronal and axial planes, and axial T2w fast spin echo-inversion recovery (FSE-IR) for white matter according to the anterior commissure—posterior commissure (AC-PC) anatomical landmarks; axial T1-weighed (T1w) 3D prepped inversion recovery fast spoiled gradient echo (brain volume acquisition, BRAVO); sagittal T2w 3D fluid-attenuated inversion-recovery with fat saturation (3D FLAIR fat sat); 3D susceptibility-weighted angiography (SWAN), diffusion weighted images (DWI) with *b* values of 0 and 1000 s/mm^2^; and 3D BRAVO and axial T1 FSE acquired after the i.v. administration of 0.1 mml/kg of gadobutrol (Gadovist, Bayer HealthCare) using an automatic injector (Medrad Spectris Solaris EP, Bayer Healtcare). MRI acquisition parameters are detailed on Table 1. The same protocol was repeated at 48-h, 6-months, and subsequent follow-ups (1, 2 and 5-years).

**Table 1:** MRI acquisition parameters used for screening and follow-up studies with 1.5 T MRI scanner.

Sequences	Slice thickness (mm)	Gap	TR (ms)	TE (ms)	IT (ms)	Matrix	NEX	FOV (cm)
Sagittal T2w FRFSE^a^	2	0	5269	97	n.a	224 × 224	5	24 × 24
Coronal T2w FRFSE^a^	2	0	4387	108	n.a	320 × 288	5	24 × 24
Axial T2w FEFSE^a^	2	0	4380	108	n.a	320 × 288	5	24 × 24
Axial T2w FSE-IR^a^	2	0	4500	40	300	320 × 288	1	24 × 24
Axial T1w 3D IR BRAVO	1	0	12.4	5.2	450	256 × 256	1	25.6 × 25.6
Sagittal T2w 3D FLAIR Fat Sat	1.2	0	6000	124.6	1866	224 × 224	1	26 × 26
EP-DWI	5	1	7000	Shortest	n.a	128 × 128	1	24 × 24
Axial 3D SWAN	3	0	78.5	50	n.a	288 × 224	1	23 × 23
Axial T1w FSE	5	1	475	Shortest	n.a	256 × 192	2	24 × 24

*TR* repetition time, *TE* echo time, *TI* inversion time, *NEX* number of excitations, *FOV* field of view, *FRFSE* fast recovery fast spin echo, *FSE* fast spin echo, *IR* inversion recovery, *BRAVO* brain volume acquisition, *FLAIR Fat Sat* fluid-attenuated inversion-recovery with fat signal saturation, *EP* echo planar, *DWI* diffusion weighed imaging, *SWAN* susceptibility-weighted angiography.

^a^Basal ganglia coverage.

### CT technique

Screening CT imaging were acquired using a multidetector 16-channel CT scanner (BrightSpeed, GE Medical Systems). The protocol was as follow: sequential acquisition with no gantry inclination; tube voltage 120 kV; tube current 220 mAs; slice thickness 1.25 mm; spacing 0 mm; reconstruction kernel bone plus. The SDR was calculated using the ExAblate workstation (INSIGHTEC Ltd.).

### Procedure details

At our site tcMRgFUS thalamotomies were performed using a focused ultrasound (FUS) equipment (ExAblate 4000; INSIGHTEC Ltd.) integrated with the same MR unit used for screenings. The FUS equipment is composed by a 30 cm wide hemispheric 1024-elements phased-array transducer operating at 650 kHz. All the procedures were performed by a single primary operator who had the full control of the workstation, in collaboration with a dedicated trans-disciplinary team (including neuroradiologists, neurologists, neurosurgeons, and medical physicists).

Detailed description of tcMRgFUS thalamotomy have been extensively described in prior reports^2,31,33^. Importantly, to avoid the passage of the HI-FU beams through sensitive structures and thus harmful non-targeted heating, no-pass regions (NPRs) must be marked on coregistered screening CT and MRI examinations. Typical NPRs were placed during treatment planning on CT images outlining frontal sinuses, choroid plexus and intracranial calcifications^2,31^. On treatment day, additional NPRs were marked if needed (folds of the silicon membrane as well as nevi, skin scars, or other skin defect revealed after patient’s head was shaven that may interfere with HI-FU beams). More NPRs were placed according to the evidence of preoperative findings on screening CT and MRI, considering the risk of ultrasound absorption and related unwanted heating. In our practice we calculated the maximum diameter of each incidental imaging finding on the MRI sequence in which it was best depicted. A volumetric NPR was then placed with the treatment workstation (ExAblate, INSIGHTEC Ltd.) eventually using MRI volume coordinates if the depicted imaging finding was not appreciable on planning T2-w FRFSE images. The NPRs prevent unwanted exposures by turning off all the transducer elements whose beam would pass through it. Notably, at least 700 elements should be active to allow the treatment^2,31^.

### Imaging analysis

Preoperative CT and MRI studies were retrospectively evaluated by two radiologists (C.G. with 15 and R.C. with 5 years of experience, respectively). Imaging studies were reviewed using an independent workstation equipped with Horos (a free and open source code software program that is distributed free of charge under the LGPL license at Horosproject.org). Preoperative incidental imaging findings were defined as any lesion, mass, or potentially relevant abnormality for the tcMRgFUS procedure detected on CT and MRI screening examinations. Findings as anatomical variants of the sinus cavities, or intracranial calcifications were not included as these structures are routinely marked with NPRs in all patients during treatment planning. The presence and location of imaging findings on CT and MRI in each patient was registered as well as the MRI sequences where the findings could be better identified and characterized. For SWAN sequences only the presence of hypointense lesions non-compatible with calcifications on corresponding CT images were recorded.

All patients treated with tcMRgFUS were reviewed using the dedicated workstation (ExAblate, INSIGHTEC Ltd.) in order to evaluate the additional number of transducers that were shut off to prevent HI-FU transmission due to the presence of above findings marked as NPRs. For the shut off transducers, it was only considered the number of elements due to the specific preoperative findings detected. This latter is related to the NPRs volume and location. Notably, even if the NPRs were placed below the plan passing through the treatment target, they were recorded because these may result in elements being shut off if crossed by far field HI-FU beams^31^. Additional treatment parameters such as SDR, skull area exposed to the HI-FU beams, and number of active transducers elements were registered in treated patients.

Based on the review of all tcMRgFUS procedures and trans-disciplinary discussion, preoperative findings were classified in three different groups according to their relevance (in terms of energy absorbing structures or sensitive tissues) for the eligibility and treatment planning:

- Group 1: not significant or scarcely significant, if they should not influence the treatment planning or require any NPRs.
- Group 2: potentially significant, including findings that may impede HI-FU transmission and require placement of NPRs on the planning software, through which no acoustic energy should be delivered, or may represent potential treatment contraindications.
- Group 3: significant, if they represent a clinically relevant incidental finding requiring urgent or immediate referral thus an absolute contraindication for the treatment.

### Statistical analysis

Continuous variables were expressed as mean and standard deviation (SD) while categorical variables as numbers and percentages. The Mann–Whitney U test was performed to assess the difference of the number of preoperative findings in patients < 65 years compared to patients ≥ 65 years (cutoff based on the mean age of the included patients) and the difference in number of treatment elements in patients with or without group 2 findings. Correlation between the number of preoperative findings and patients’ age was calculated using the Spearman’s rank correlation coefficient (Spearman’s rho).

Statistical significance level was set at p < 0.05. Statistical analysis was conducted by using SPSS software (Version 25.0. Armonk, NY, USA: IBM Corp).

### Ethical approval

All procedures performed in the studies involving human participants were in accordance with the ethical standards of the institutional and/or national research committee and with the 1964 Helsinki Declaration and its later amendments or comparable ethical standards.

### Informed consent

Informed consent was obtained from all individual participants included in the study.

## Results

### Population

**Figure 1:**
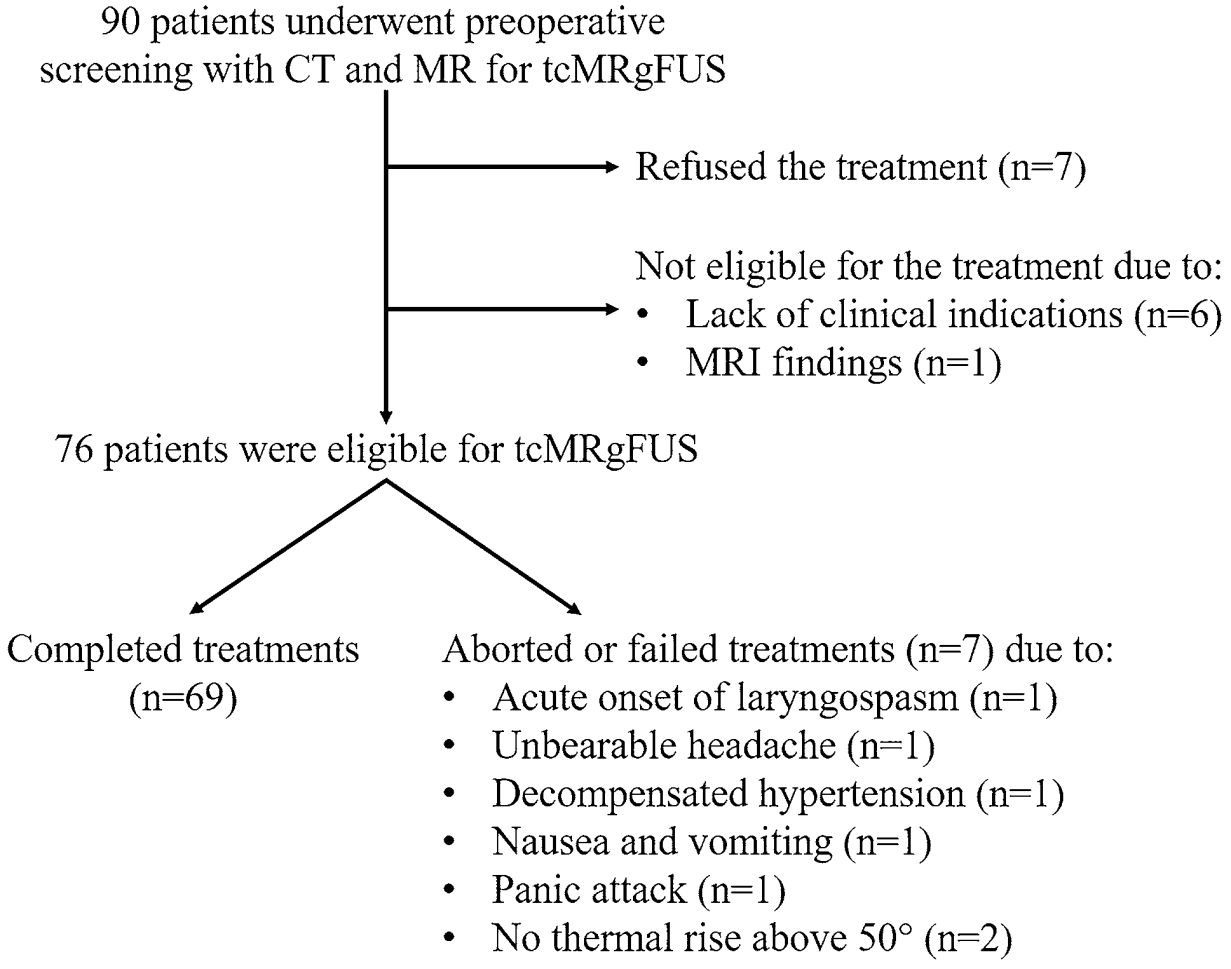
Flow diagram of the screening and treatment population.

Ninety patients (67 men and 23 women, mean age at the time of screening: 64.7 ± 12.1 years, range 23–86 years) underwent preoperative CT and MRI before tcMRgFUS (Table 2). Indications for treatment were either essential tremor (n = 70, 77.8%), idiopathic asymmetrical tremor-dominant Parkinson’s disease (n = 15, 16.7%), multiple sclerosis (n = 3, 3.3%; two of which with essential tremor comorbidity), or neuropathic pain (n = 2, 2.2%). After multidisciplinary evaluation, additional 14 patients were considered not eligible for the treatment due to clinical indications (i.e. not definitive diagnosis of movement disorder or too subtle symptoms, n = 6) or MRI findings (n = 1) (Fig. 1). In addition, seven patients refused to be treated after completing the screening tests and being extensively informed of benefits and potential risks of the treatment. Therefore, 76 patients were finally considered eligible and provided full consent for the tcMRgFUS procedure. Treatment was completed successfully placing a lesion in the targeted area in 69 (90.7%) patients (54 men and 15 women, mean age 65.5 ± 11.5 years, range 26–86 years). Five (6.6%) procedures were aborted before realizing any sonication or placing any permanent lesion (due to acute onset of laryngospasm, unbearable headache, decompensated hypertension, nausea and vomiting, panic attack, respectively) and two (2.7%) procedures failed because it was not possible to achieve therapeutic thermal rise (above 50° during high-energy sonications) due to unfavorable skull characteristics (SDR of 0.35 and 0.38, respectively). Parameters of the treated patients were (mean ± SD): SDR, 0.50 ± 0.09; number of elements, 930.4 ± 47.9; skull area, 344.5 ± 27.2.

**Table 2:** Characteristics of the study population.

Characteristics	Number
Patients	90
**Sex**	
Males	67 (74.4%)
Females	23 (25.6%)
**Age (years)**	
Mean ± SD	64.7 ± 12.1
Range	23–86
**Tremor etiology**	
Essential tremor	70 (77.8%)
Parkinson tremor	15 (16.7%)
Multiple sclerosis	3 (3.3%)
Neuropathic pain	2 (2.2%)
**SDR**	
Mean ± SD	0.49 ± 0.09
Range	0.27–0.74

*SDR* skull-density ratio, *SD* standard deviations.

### Prevalence of preoperative findings

**Figure 2:**
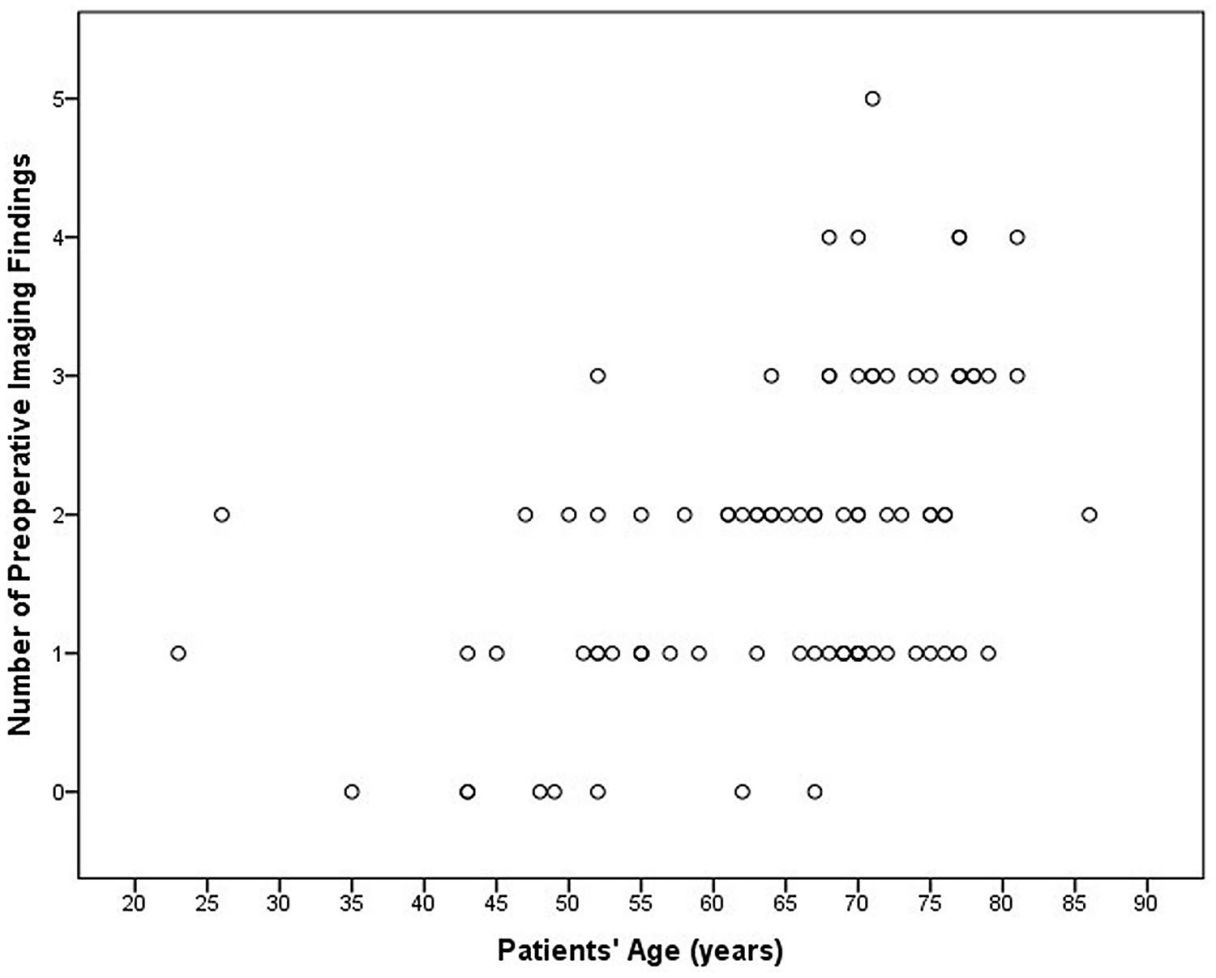
Correlation between number of preoperative neuroradiological findings in each patients and patient’s age.

A total of 146 preoperative imaging findings were detected in the screening population (Table 3). Preoperative findings were present in 79 (87.8%) patients, while 11 (12.2%) did not have any incidental imaging finding. Overall, 33 (36.7%) patients had a single finding, 29 (32.2%) had two findings, 13 (14.5%) had 3 findings, and 4 (4.4%) had 4 findings. There was a statistically significant positive correlation between the number of preoperative findings in each patient and the patients’ age (rho = 483, p < 0.001) (Fig. 2), particularly patients older than 65 years had significantly higher number of preoperative findings compared to patients < 65 years (1.8 ± 1.0 vs 1.1 ± 0.8; p = 0.001).

**Table 3:** Number and prevalence of preoperative findings in candidates for tcMRgFUS.

Preoperative findings	Number (%)	Prevalence	CT detected	Group
Gliotic foci	57 (39.0%)	63.3%	0 (0%)	1
Chronic hypoxic-ischemic encephalopathy	27 (18.5%)	30.0%	0 (0%)	1
SWAN hypointense lesion	14 (9.5%)	15.5%	0 (0%)	2
Aracnoidocele	12 (8.2%)	13.3%	0 (0%)	1
Developmental venous anomaly	5 (3.4%)	5.5%	0 (0%)	1
Lacunar infarct	5 (3.4%)	5.5%	0 (0%)	1
Multiple sclerosis lesion (not active)	3 (2.0%)	3.3%	0 (0%)	2
Cavernoma	3 (2.0%)	3.3%	1 (33.3%)	2
Meningioma	2 (1.4%)	2.2%	1 (50.0%)	2
Cavum Vergae	2 (1.4%)	2.2%	2 (100%)	1
Subdural hygroma	2 (1.4%)	2.2%	0 (0%)	1
Pineal Cyst	2 (1.4%)	2.2%	0 (0%)	1
Bone or subcutaneous lesions	2 (1.4%)	1.1%	2 (100%)	2
Multiple sclerosis lesion (active)	1 (0.7%)	1.1%	0 (0%)	2
Subdural hematoma	1 (0.7%)	1.1%	1 (100%)	3
Metallic foreign body (sliver in the frontal subcutaneous tissue)	1 (0.7%)	1.1%	1 (100%)	2
Asymmetry of the lateral ventricles	1 (0.7%)	1.1%	1 (100%)	1
Basilar artery vascular ectasia	1 (0.7%)	1.1%	0 (0%)	1
Cavum septum pellucidum	1 (0.7%)	1.1%	1 (100%)	1
Choroidal fissure cysts	1 (0.7%)	1.1%	0 (0%)	1
Mega cisterna magna	1 (0.7%)	1.1%	0 (0%)	1
Acoustic schwannoma	1 (0.7%)	1.1%	0 (0%)	1
Temporal bone lesion (petrous apicitis)	1 (0.7%)	1.1%	1 (100%)	1

Gliotic foci were the most common neuroradiological finding (n = 57, 39.0% of all findings), with an overall prevalence of 63.3%. Other common preoperative findings were chronic hypoxic-ischemic encephalopathy (n = 27, 18.5%), SWAN hypointense lesions (n = 14, 9.5%), and aracnoidocele (n = 12, 8.2%).

### Relevance of preoperative findings

No complications related to the reported preoperative imaging findings were registered in our population at intraoperative imaging or subsequent MRI follow-ups.

**Figure 3:**
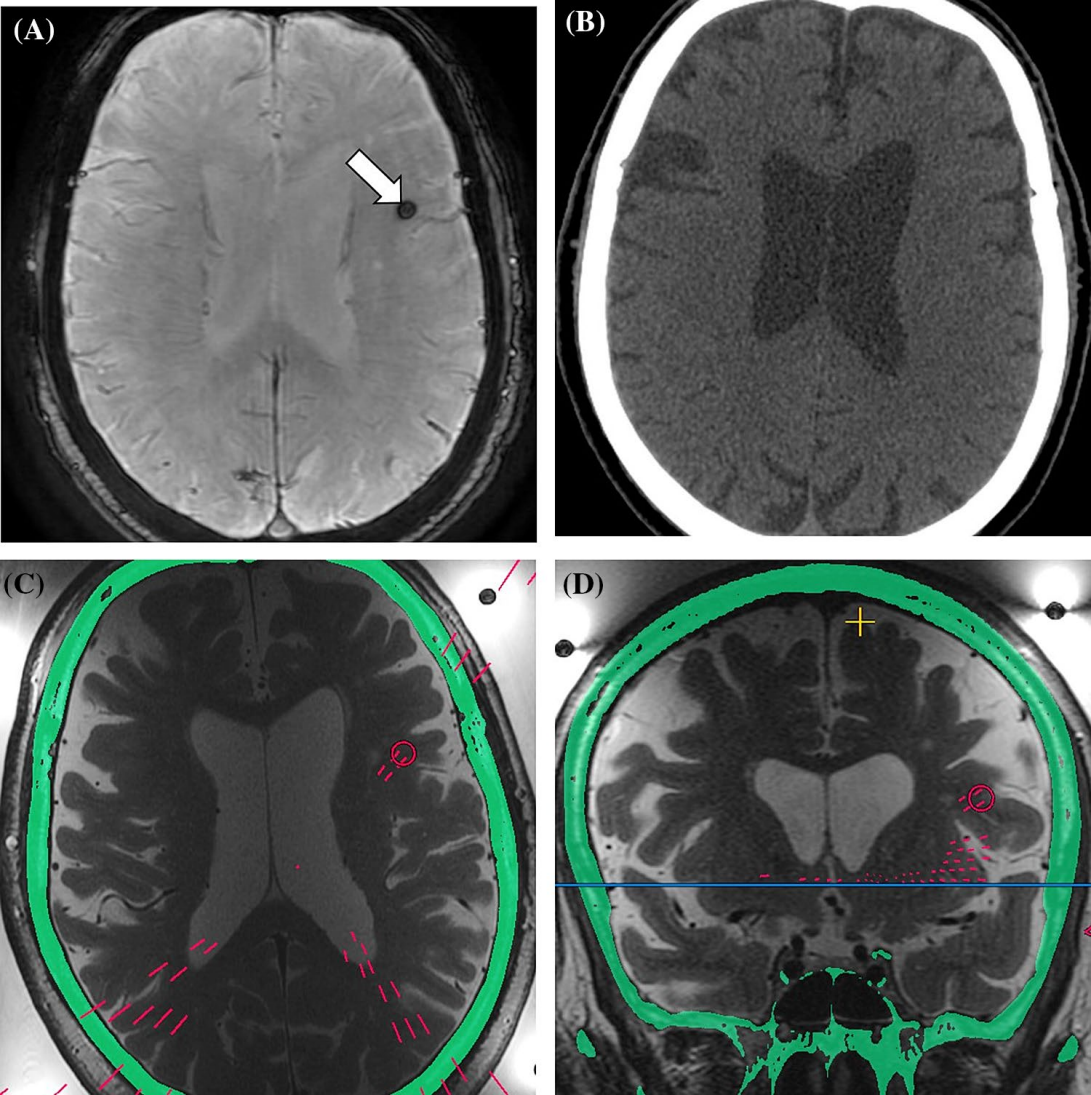
70-year-old man with essential tremor. Preoperative SWAN sequence detects a hypointense lesion in the left frontal lobe (**A**, arrow), not corresponding to calcification on CT image (**B**). Axial and coronal intraoperative T2-weighted FRFSE show the no-pass region that was placed during treatment planning [red circles in (**C**) and (**D**)], blocking 4 additional elements (4.6% of the total elements turned off).

**Figure 4:**
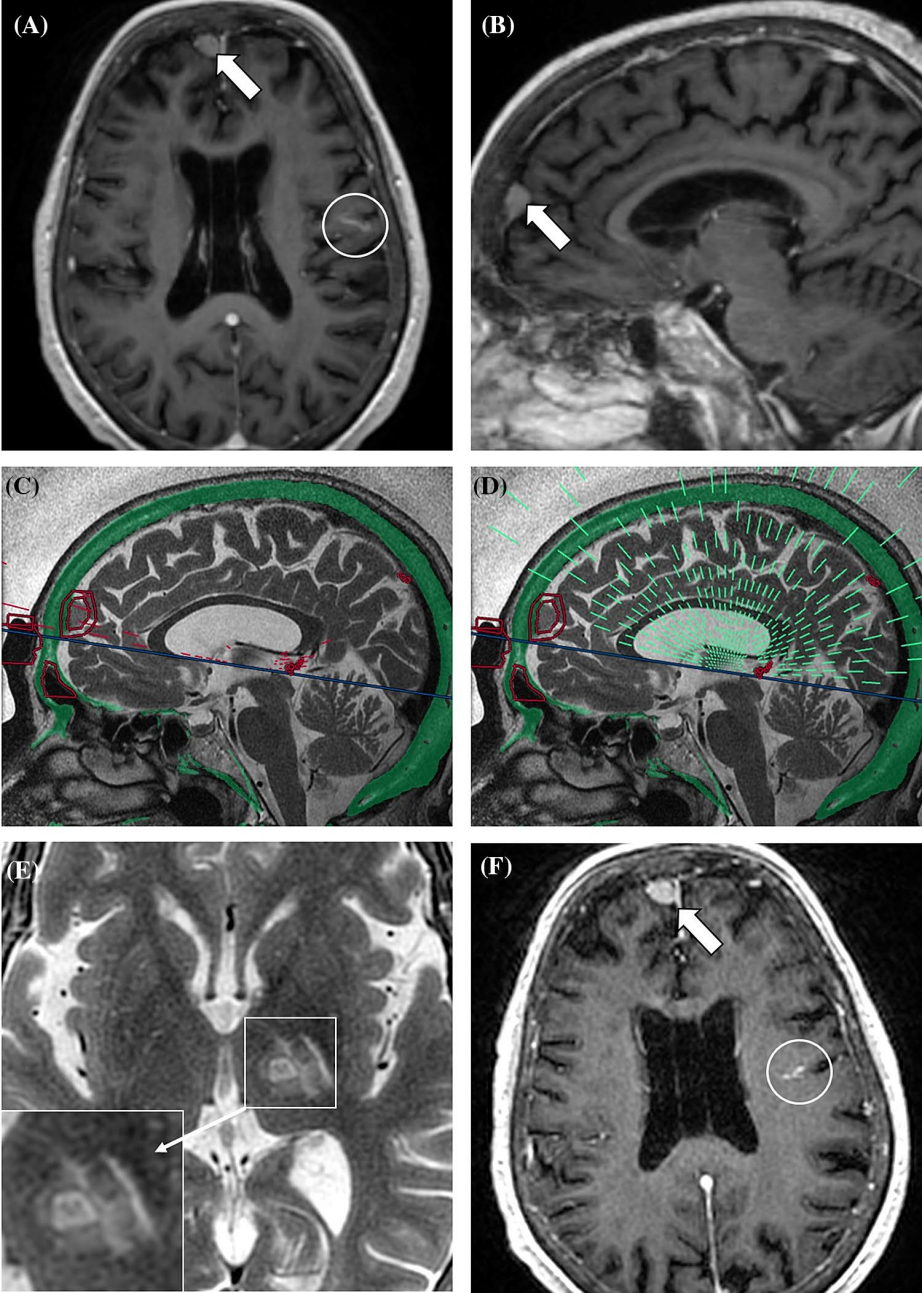
81-year-old woman with essential tremor. Preoperative axial (**A**) and sagittal (**B**) multiplanar reconstructions (3 mm thick, average algorithm) from post-contrast T1w 3D BRAVO demonstrate a 11 mm extra-axial lesion compatible with meningioma (arrows). A developmental venous anomaly in the left fronto-parietal region is also present (white circle). Sagittal intraoperative T2-weighted FRFSE images show no-pass regions (red circles) with blocked [(**C**) red lines] and active [(**D**) green lines] elements. A total of 13 additional elements (11.6% of all blocked elements) were turned off due to the presence of the meningioma. 48-h follow-up axial T2-weighted FRFSE sequence (**E**) shows the FUS-placed lesion in the left nucleus ventralis intermedius. Post-contrast (**F**) image demonstrates no changes in the meningioma (arrow) or developmental venous anomaly (white circle).

With regard of the group classification, 119 (81.5%) preoperative findings belonged to group 1 and they did not require any modification in the treatment planning. Twenty-six (17.8%) neuroimaging findings in 23 (25.6%) patients were classified as group 2, since they may represent potential contraindications or may require the placement of NPRs during procedure planning, through which no acoustic energy should be delivered. SWAN hypointense lesions were the most common finding in the group 2 (Fig. 3), with a prevalence of 16.7%. Three (3.3%) patients had multiple sclerosis lesions (active plaque revealed at screening MRI in one case), of which the two with essential tremor comorbidity were successfully treated (without complications neither evidence of disease activity nor progression in the follow-up period) while one procedure was aborted (acute onset of laryngospasm before starting the treatment in the patient who had the active plaque at screening MRI). Brain cavernomas were detected in three (3.3%) patients at preoperative MRI and they were located below the AC-PC plane in one patient (cerebellar hemisphere) and above in two patients. Two patients with a cavernoma located in the left cingulate cortex and in the left cerebellar hemisphere refused the treatment after being fully informed about the possible procedural risks. A 68-year-old man with a 5 mm hypointense SWAN lesion in the frontal lobe, very likely to be a small cavernoma, underwent tcMRgFUS without procedural complications. Two (2.2%) patients were discovered with lesions compatible with meningioma, including a 66-year-old woman with 6 mm entirely calcified meningioma visible on CT imaging and with a dural base in the left temporal bone and an 81-year-old woman with an 11 mm meningioma with a dural base in the right frontal bone. Both patients were successfully treated without complications (Fig. 4). An unexpected millimetric metallic foreign body was detected in a 67-year-old man who performed CT screening after MRI. After collegial discussion, aware that the procedure could have been aborted in case of artifacts on MRI sequences used for monitoring the thermal rise during sonications, the patient was successfully treated with tcMRgFUS. A right subacute on chronic subdural hematoma was discovered in a 55-year-old man with history or chronic headache, and the patient was considered ineligible for the treatment (group 3); this patient would have been excluded anyway from treatment because of a way to low average SDR score (0.27).

Additional NPRs placed in patients with group 2 findings blocked a mean number of 4.6 (range 0–16) elements, accounting for a mean of 6.5% (range 0–24.2%) of all blocked elements during the treatment planning in patients undergoing tcMRgFUS procedure (Figs. 3 and 4). When considering routinely placed NPRs and elements blocked by preoperative imaging findings, there was no statistically significant difference in the final number of active elements in treated patients with or without group 2 findings (945.6 ± 34.9 vs 925.7 ± 50.6; p = 0.235).

### MRI sequences for the characterization of preoperative findings

A retrospective analysis was conducted in order to evaluates the role of each MRI sequences in the detection of imaging findings according to the subgroup classification. T2w FRFSE and 3D-FLAIR characterized 118 (99.1%) and 116 (97.5%) group 1 imaging findings, respectively. However, only 5 (19.2%) and 6 (23.1%) group 2 imaging findings could be characterized by using T2w FRFSE or 3D-FLAIR, respectively. Other group 2 imaging findings were observed with additional sequences, such as SWAN (n = 18, 69.2%, including 14 microbleeds, 3 cavernomas and 1 calcified meningioma, the latter already appreciable on screening CT) and T1w post-contrast images (n = 3, 11.5%, including one cavernoma, one meningioma, and one active plaque in a multiple sclerosis patient). T1w post-contrast images identified also one acoustic schwannoma and five developmental venous anomalies (one of which associated with a cerebellar cavernoma). Five group 2 imaging findings could be detected and characterized by CT, including one cavernoma, one calcified meningioma, two bone and subcutaneous lesions, and one metallic foreign body. The subdural hematoma in the only patient with group 3 finding could be detected with all MRI sequences and CT images.

## Discussion

Candidates to tcMRgFUS undergo pretreatment neuroimaging screening with CT and MRI which may lead to unexpected detection of incidental imaging findings. In this study we investigated the prevalence and significance of preoperative neuroradiological findings in a prospectively-enrolled cohort of candidates to tcMRgFUS. In our study, preoperative neuroimaging findings were present in 87.8% of patients, with a significantly positive correlation with patients’ age (p < 0.001). Most of the preoperative findings (81.5%) were classified as group 1, without significant implications for the treatments. Particularly, gliotic foci and signs of chronic hypoxic-ischemic encephalopathy were the most common imaging findings in our population (39.0% and 18.5%, respectively), and they typically did not require NPRs placement since they do not affect HI-FU transmission, even if potentially clinically relevant^34^. Only one patient with a hemispheric subdural hematoma, incidentally discovered on neuroradiological screening examinations, was classified in the group 3 and therefore excluded from the procedure.

TcMRgFUS allows high energy deposition in a precise target (i.e. nucleus ventralis intermedius in patients with tremor) inducing coagulation necrosis without thermal injury to the non-target surrounding tissues. Prior evidences have reported a high success rate and safety of tcMRgFUS, with most of adverse events being mild and transient^21,35–38^. Adverse events may be related to HI-FU passage (i.e. as headache, vertigo, nausea and vomiting) or to the ultrasound ablation thalamotomy and subsequent edema surrounding the ablated area (i.e. temporary paresthesia or ataxia)^21,38^. Several factors have been investigated and correlated with the rate of successful treatments. The skull is the most critical barrier to the ultrasound delivery. Skull density ratio and skull volume are well-known factors affecting the thermal rise^32,39^. Prior studies have also reported a correlation between the number of elements on and thermal rise during sonications^39^. As a consequence, a total number of at least 700 effective elements and a skull area of at least 250 cm^2^ are mandatory conditions for the treatment^2,31^. Preoperative neuroimaging findings may have a significant impact for patients eligibility to tcMRgFUS and may necessitate to reduce a large number of available FUS elements below the treatment cutoff.

In our experience, no complications related to the reported preoperative imaging findings were observed at intraoperative imaging or subsequent follow-ups. However, 26 (17.8%) neuroimaging findings in 23 (25.6%) patients were classified as group 2, with potential treatment implications. Among them, most of group 2 findings were SWAN hypointense lesions, not corresponding to calcifications on CT, accounting with a prevalence of 16.7% in our population. SWAN hypointense lesions may represent areas of tissue inhomogeneity and interfaces which could potentially lead to cavitation phenomena and direct damage to tissue^40^. In our experience we recommend placement of NPRs in these lesions to impede the ultrasound delivery and mitigate the potential risk of potential unwanted heating. Overall, the presence of group 2 findings resulted in an additional mean reduction of 6.2% elements. However, these additional NPRs did not significantly decrease the number of active FUS elements compared to the patients without group 2 findings and none of them reduced the active FUS elements below the threshold of 700, which is the cutoff that allow the treatment^2,31^. Further studies are needed to test the safety of tcMRgFUS in patients with specific neuroradiological findings and the risk of thermal/cavitation damage associated with these lesions.

Only few prior reports of tcMRgFUS treatment exist in patients with specific neuroradiological conditions^36,41,42^ or with intraoperative imaging^43^. Yang et al.^41^ reported a tcMRgFUS treatment in a patient with essential tremor and ventricular shunt for normal pressure hydrocephalus requiring 6% or ultrasound elements being shut off. Recently, Máñez-Miró et al.^42^ reported the first successful tcMRgFUS thalamotomy in a patient with multiple sclerosis-associated tremor. In our study, three patients had multiple sclerosis and one of them had enhancing plaque during preoperative imaging that required reassessment after medical therapy. These patients were classified as group 2 since they should be carefully selected considering the treatment benefits and potential risk of non-reversible ablation in a brain with impaired compensatory mechanism due to multiple sclerosis plaques^42^. In our population two patients with meningioma (one of them entirely calcified) and one patient with a cavernoma (in the frontal lobe) were successfully treated with tcMRgFUS without complications, while two patients with a cavernoma of the left cingulate cortex and of the cerebellar hemisphere refused the treatment after being fully informed about procedure risks and benefits. To the best of our knowledge this is the first report dedicated to the role of incidental imaging findings identification in patients underwent to tcMRgFUS thalamotomy.

Nowadays there is no consensus regarding the optimal preoperative screening protocol. Only few prior studies have detailed the sequences acquired in preoperative imaging^7,44–46^. In our University Hospital, T2w FRFSE is a key sequence for treatment planning, but it may identify only a limited number of preoperative findings and furthermore these sequences are acquired with a coverage limited to the basal ganglia region. In our study, most of the group 2 findings were identified on SWAN sequences. This sequence is particularly helpful in the identification of small hypointense foci without correlate on CT, likely corresponding to hemosiderin deposits that require NPRs placement. Thus, including susceptibility-weighted sequences in MRI screening protocol is highly recommendable. Post-contrast imaging had an also an important role in the preoperative workup and allowed to identity lesions as small meningioma, active plaque in multiple sclerosis, acoustic schwannoma, and developmental venous anomalies.

Our study has a major limitation that needs to be acknowledge. In our population, only patients with a confirmed diagnosis and confirmed clinical indication at neurological and neurosurgical screening undergo CT and MRI screening examinations. Therefore, our findings may not be representative of all patients with essential tremor, Parkinson disease, neuropathic pain, or other neurodegenerative diseases. A further limitation of our study concerns the classification in the three groups of imaging findings on which this study is based. This classification if of course not comprehensive of all neuroradiological findings that could be found in a candidate to this cutting-edge non-invasive procedure but at the same time it includes the most common which may require specific technical measures. For example, the finding of large benign tumor lesions (i.e.: meningioma of the convexity for near field exposure or of the skull base far-field exposure), severe metal deposits in the basal ganglia or other neuroradiological findings not evaluated in the current paper will require further studies and insights to clarify the safety and feasibility of these procedures in these borderline conditions.

In conclusion, preoperative neuroradiological findings are frequently encountered in candidates to tcMRgFUS and their identification may require the placement of additional no-pass regions. In our experience, the additional findings did not significantly affect the number of FUS elements. Nevertheless, marking SAWN hypointense lesions or other group 2 lesions as no pass regions should be considered mandatory to avoid unwanted heating and related possible damage to untargeted brain structures. Considering the growing trend of tcMRgFUS procedures for functional neurological disorders and the expanding number of experimental applications, we suggest that more and more attention should to be given to potentially harmful imaging findings to offer this therapeutic option to an ever-increasing number of patients.
